# Aspirin therapy after heart transplantation is associated with higher myocardial perfusion reserve assessed by cardiovascular magnetic resonance imaging

**DOI:** 10.1016/j.jhlto.2026.100592

**Published:** 2026-05-10

**Authors:** R. Jablonowski, I.H. Löfman, H.B. Andersson, G. Gjesdal, C. Fogarasi, P. Kellman, M. Melin, H. Engblom, H. Arheden, M. Carlsson, J. Nickander, O.Ö. Braun

**Affiliations:** a Clinical Physiology, Department of Clinical Sciences Lund, Lund University, Skane University Hospital, Lund, Sweden; b Cardiology, Department of Clinical Sciences Lund, Lund University, Skane University Hospital, Lund, Sweden; c Department of Molecular Medicine and Surgery, Karolinska Institute, Stockholm, Sweden; d National Heart, Lung, and Blood Institute National Institutes of Health, Bethesda, MD; e Department of Laboratory Medicine, Karolinska Institutet, Huddinge, Sweden; f Department of Medicine, Karolinska Institute, Solna, Stockholm, Sweden; g Department of Clinical Science and Education, Karolinska Institutet, Södersjukhuset, Stockholm, Sweden

**Keywords:** Heart transplant, Cardiovascular magnetic resonance, Myocardial perfusion, Aspirin, Cardiac allograft vasculopathy

## Abstract

**Background:**

Cardiac allograft vasculopathy (CAV) is one of the major long-term complications in patients after heart transplantation (HTx) limiting allograft function and longevity. CAV can be studied non-invasively by evaluating myocardial perfusion (MP). Aspirin is re--ed after Htx, but it is unclear whether it affects the development of CAV. The aim of this study was to investigate the association between aspirin therapy and MP, as a marker of CAV, using cardiovascular magnetic resonance (CMR).

**Methods:**

We consecutively included HTx patients from 2 centers in Sweden who were examined with CMR and coronary angiography. Patients were categorized by the presence or absence of aspirin therapy. As a part of routine surveillance, quantitative short-axis perfusion maps were acquired at rest and during stress hyperemia. A global average MP was calculated at rest and stress and myocardial perfusion reserve (MPR) was defined as the ratio between stress and rest MP. Cine images and T1 maps were also acquired.

**Results:**

A total of 137 patients (*n* = 107 aspirin) were included in the study. Resting myocardial perfusion was lower in HTx recipients receiving aspirin therapy compared with those not receiving aspirin (1.0 ± 0.3 vs 1.2 ± 0.4 ml/min/g; *p* = 0.003); stress myocardial perfusion was higher in patients treated with aspirin (2.6 ± 0.8 vs 2.2 ± 1.0 ml/min/g; *p* = 0.037). Consequently, myocardial perfusion reserve (MPR) was significantly greater in patients receiving aspirin compared with those not on aspirin therapy (2.5 ± 0.7 vs 1.9 ± 0.8; *p* < 0.001). In both univariable and multivariable linear regression analyses, aspirin therapy remained independently associated with higher MPR after adjustment for sex, CAV grade, time from transplantation to CMR, diabetes, statin therapy, and everolimus use.

**Conclusion:**

In this retrospective study, aspirin therapy in heart transplant patients was independently associated with higher myocardial perfusion reserve.

## Background

Cardiac allograft vasculopathy (CAV) is among the most common long-term complications following heart transplantation and remains a leading cause of late graft failure and mortality.^1^, ^2^, ^3^ Unlike native coronary artery disease, CAV is characterized by diffuse, concentric intimal proliferation affecting both epicardial coronary arteries and the microcirculation.^4^, ^5^, ^6^ The pathogenesis of CAV is multifactorial and includes immune-mediated endothelial injury, metabolic insults, ischemia-reperfusion injury, donor-specific antibodies, and chronic inflammation.^1^, ^4^, ^6^ Over time, progressive luminal narrowing and microvascular dysfunction impair coronary blood flow and myocardial perfusion, ultimately compromising allograft function and contributing to adverse clinical outcomes.^1^, ^2^, ^3^, ^6^ However, the potential role of aspirin in preventing CAV remains less clear. Although aspirin is widely used after heart transplantation and current International Society for Heart and Lung Transplantation (ISHLT) guidelines state that routine early aspirin use may be considered for prevention of CAV, the supporting evidence remains limited and is based primarily on observational studies rather than randomized trials.^4^ Prior observational analyses have reported that early aspirin exposure after heart transplantation is associated with a lower risk of developing moderate-to-severe CAV and may also be associated with improved long-term outcomes.^7^, ^8^, ^9^ However, no completed randomized controlled trial has established whether aspirin causally prevents CAV progression or improves graft vascular function. Therefore, further studies using sensitive markers of graft vascular health are warranted.

Cardiovascular magnetic resonance (CMR) imaging has emerged as a tool for detecting both epicardial and microvascular abnormalities in CAV,^5^, ^10^, ^11^, ^12^, ^13^, ^14^ and recent work has also highlighted the complementary roles of non-invasive and invasive imaging strategies in the evaluation of CAV.^15^ Pixel-wise perfusion mapping enables absolute quantification of myocardial blood flow and myocardial perfusion reserve (MPR).^16^, ^17^, ^18^ Recent studies have demonstrated the feasibility of stress quantitative CMR and its ability to reliably detect different stages of CAV, with an MPR cutoff of 2.2 demonstrating high sensitivity for identifying significant vasculopathy.^19^, ^20^

The aim of this study was to evaluate whether aspirin therapy is associated with differences in myocardial perfusion using quantitative CMR perfusion in adult heart transplant recipients compared to those with no aspirin therapy.

## Methods

### Study design and setting

This study was designed as a retrospective observational cohort study including consecutive adult heart-transplant recipients who underwent CMR imaging and invasive coronary angiography at 2 Swedish transplant centers (Skåne University Hospital in Lund and Karolinska University Hospital in Stockholm). During the later part of the study period, stress CMR was introduced as part of routine post-transplant surveillance at the participating centers, with a planned examination at approximately 1 year post transplant. However, as this protocol was implemented during the study period, not all patients underwent CMR at the 1-year follow-up. In patients with more than 1 post-transplant CMR examination, the first available study fulfilling the inclusion criteria was used for analysis. Examinations performed because of suspected or ongoing rejection were excluded. Clinical data, medication history, angiographic findings, and CMR parameters were extracted from the electronic medical records and imaging archives.

### Study population

All adult heart-transplant recipients who underwent surveillance CMR imaging between 2018 and 2023 were screened for inclusion. Patients were eligible if they had also completed an invasive coronary angiography as part of scheduled post-transplant follow-up.

Patients were not referred for CMR if they had severe renal impairment (estimated glomerular filtration rate <30 ml/min/1.73 m²), contraindications to gadolinium contrast or vasodilator stress agents (adenosine or regadenoson), or remaining pacemaker leads. After applying these criteria, the final cohort consisted of 137 patients who met all inclusion criteria and had complete imaging and clinical data.

Ethical approval was granted by the regional ethics committee in Lund (applications 2004/741 with amendment 2018/948 and 2013/319) and by the Swedish Ethical Review Authority (Dnr 2013/900, 2015/248, 2018/431, 2023–06876–01, and 2023–01479–01). All procedures complied with the ISHLT Ethics Statement, and written informed consent was obtained from all participants.

### Baseline clinical data and medication assessment

Baseline clinical information included age, sex, anthropometric data, blood pressure, years from transplantation to imaging, renal function, lipid profiles, glycemic indices, inflammatory markers, NT-proBNP, troponins, and other laboratory measurements.

Medication histories were adjudicated through detailed review of electronic chart notes. The standard immunosuppressive protocol consisted of induction with 3 doses of antithymocyte globulin (ATG), followed by maintenance therapy with prednisolone, tacrolimus, and mycophenolate mofetil (MMF). Everolimus is not used routinely but may be introduced as a calcineurin inhibitor (CNI)—sparing strategy in cases of impaired renal function, most commonly within the first 6 months after transplantation at the discretion of the treating physician. In addition, everolimus may be introduced later in the post-transplant course in patients with early development of CAV, typically within the first 3 years after transplantation. Statins and antihypertensives were also recorded. Aspirin exposure was defined as therapy with 75 mg once daily at the time of CMR. The protocol re--ation for aspirin did not change during the study. All medications listed in Table 1 were recorded as part of routine clinical documentation at the time of CMR.

**Table 1:** Baseline Characteristics

		No aspirin	Aspirin	*p* value
Number of patients		30	107	
Reason for transplantation	DCM	11 (37%)	45 (42%)	0.86
	IHD	2 (15%)	7 (13%)	
Age at transplantation, median (IQR)		49 (30, 62)	47 (33, 56)	0.59
Sex	Male	21 (70%)	71 (66%)	0.71
	Female	9 (30%)	36 (34%)	
Weight (kg), median (IQR)		83 (69, 92)	81 (69, 90)	0.96
Height (cm), median (IQR)		172 (165, 183)	175 (168, 182)	0.38
Years from HTx to CMR, median (IQR)		8 (2, 12)	4.5 (1.2, 9.9)	0.20
CAV grade	0	12 (40%)	59 (55%)	0.28
	1	16 (53%)	40 (37%)	
	2-3	2 (7%)	8 (7%)	
SBP (mmHg), mean (SD)		132 (15)	129 (13)	0.37
DBP (mmHg), mean (SD)		81 (10)	80 (12)	0.49
Creatinine (µmol/l), mean (SD)		107 (45)	93 (29)	0.05
Hemoglobin (g/l), mean (SD)		133 (19)	139 (17)	0.09
CRP (mg/l), mean (SD)		3.3 (5)	2.5 (3.3)	0.31
HBA1c (mmol/mol), mean (SD)		40 (8)	40 (10)	0.77
LDL (mmol/l), mean (SD)		2.3 (0.8)	2.1 (0.7)	0.33
HDL (mmol/l), mean (SD)		1.5 (0.6)	1.4 (0.4)	0.25
Triglycerides (mmol/l), mean (SD)		1.8 (0.8)	1.7 (0.9)	0.63
TnI at first CMR, mean (SD)		13 (17)	19 (33)	0.69
TnT at first CMR, mean (SD)		19 (17)	18 (21)	0.89
NT-proBNP (ng/l), mean (SD)		1106 (1381)	396 (539)	**<0.001**
CNI monotherapy		21 (70%)	80 (75%)	0.60
Everolimus monotherapy		3 (10%)	4 (4%)	0.60
Everolimus + CNI		6 (20%)	23 (22%)	0.86
Mycophenolate mofetil (MMF)		21 (70%)	93 (88%)	**0.02**
Azathioprine		3 (10%)	5 (5%)	0.31
Prednisolone		27 (90%)	87 (81%)	0.26
Statin		24 (83%)	101 (96%)	**0.01**

*P* value represents group comparisons between groups with or without aspirin treatment.

CAV, cardiac allograft vasculopathy; CMR, cardiovascular magnetic resonance; CNI, calcineurin inhibitor (Tacrolimus or Cyclosporine); DCM, dilated cardiomyopathy; HTx, denotes heart transplantation; IHD, ischemic heart disease; IQR, interquartile range; SD, standard deviation.

Statistically significant p-values are shown in bold.

### CAV grading and angiographic adjudication

Invasive coronary angiography was used to assess CAV severity. All angiographic studies were re-evaluated by an experienced cardiologist (H.B.A.) using Sectra IDS7 (Sectra Medical, Linköping, Sweden). Coronary lesions were visually graded according to the ISHLT CAV scheme: CAV0 (no significant disease), CAV1 (mild), CAV2 (moderate), and CAV3 (severe).^4^ Classification was based solely on angiographic appearance; functional parameters and intravascular imaging were not incorporated. Prior percutaneous coronary intervention (PCI) to a coronary segment was considered evidence of significant epicardial disease in that vessel and was incorporated into the overall ISHLT CAV classification together with the complete angiographic findings. Patients with prior PCI were deemed to have at least modest CAV (grade 2).

### CMR acquisition

CMR was performed on 1.5T Siemens Magnetom Aera or Sola systems (Siemens Healthineers, Erlangen, Germany). Left ventricular volumes and mass were obtained from steady-state free-precession cine images acquired during end-expiratory breath-holds. Quantitative perfusion maps were generated from dual-sequence first-pass imaging at rest and vasodilator stress following a 0.05 mmol/kg bolus of gadobutrol (Gadovist, Bayer AB, Solna, Sweden).^16^, ^18^ Stress imaging employed either adenosine infusion (140 µg/kg/min, increased to 210 µg/kg/min if needed) or a 400-µg bolus of regadenoson. For adenosine studies, stress imaging preceded rest imaging; for regadenoson, rest scans were acquired first to avoid residual hyperemia. A second 0.05 mmol/kg dose of contrast was administered for late gadolinium enhancement (LGE) imaging 10 min later. Extracellular volume (ECV) fraction was calculated from paired pre- and post-contrast T1 maps and corrected for hematocrit. Detailed CMR parameters have been published previously.^19^

### CMR analysis

Quantification of left ventricular tissue properties was performed using Segment (Medviso, Lund, Sweden)^21^ or SyngoVia (Siemens). Cine steady-state free-precession images were used to assess end-diastolic volume (EDV), end-systolic volume, and ejection fraction (EF) Inline Gadgetron software was used to generate perfusion and ECV maps. Global myocardial perfusion (MP) at rest and stress was averaged across all segments, and MPR was calculated as the stress-to-rest ratio. Resting perfusion was adjusted for hemodynamic state by calculating the rate-pressure product (RPP; resting heart rate × systolic blood pressure/10,000), yielding RPP-corrected rest perfusion and corrected MPR. Native T1 values, ECV was quantified at rest. Presence of LGE was assessed.

### Statistical analysis

Continuous variables are reported as mean ± standard deviation when normally distributed and as median with interquartile range when skewed. Categorical data are presented as counts and percentages. Group comparisons employed *t*-test for normally distributed variables, Wilcoxon rank-sum test for non-parametric variables, and Pearson’s chi-square test for categorical variables. Univariate and multivariable linear regression analyses were performed with known CAV-related variables. Statistical significance was defined as *p* < 0.05. Analyses were conducted using Stata version 18.0 (StataCorp, College Station, TX).

## Results

### Baseline characteristics

A total of 137 heart transplant recipients were included, of whom 107 were receiving aspirin therapy and 30 were not. Baseline demographic and clinical characteristics are summarized in Table 1. The 2 groups were comparable with respect to age, sex distribution, anthropometric variables, and time from transplantation to CMR. Hemodynamic parameters, including systolic and diastolic blood pressure, also did not differ significantly.

The distribution of CAV grades did not differ between groups (*p* = 0.28). In the aspirin group 6% (*n* = 6) had prior PCI, and 3% (*n* = 1) in the non-aspirin group (*p* = 0.62). Laboratory markers—including hemoglobin, C-reactive protein (CRP), HbA1c, low-density lipoprotein cholesterol (LDL cholesterol), high-density lipoprotein cholesterol (HDL cholesterol), triglycerides, troponin I and T—were similar between groups, with the exception of NT-proBNP, which was higher in the non-aspirin group (1106 ± 1381 ng/L vs 396 ± 539 ng/L, *p* < 0.001).

In the aspirin group, 75% (*n* = 80) of patients received CNI monotherapy, compared with 70% (*n* = 21) in the non-aspirin group (*p* = 0.60); everolimus in combination with CNI was used in 22% (*n* = 23) and 20% (*n* = 6), respectively (*p* = 0.86), whereas everolimus monotherapy was administered in 4% (*n* = 4) and 10% (*n* = 3), respectively (*p* = 0.17). Statin use was also more prevalent in the aspirin group (96% vs 83%, *p* = 0.011).

### Cardiac structure, function, and tissue characterization

Left ventricular volumes and systolic function were similar between aspirin and non-aspirin groups. Mean left ventricular end-diastolic volume, left ventricular end-systolic volume, left ventricular ejection fraction (LVEF), and cardiac index did not differ significantly, see Table 2. Native global T1 values and prevalence of LGE were comparable between groups. However, global extracellular volume fraction (ECV) was lower in the aspirin group (27% vs 29%, *p* = 0.04)

**Table 2:** Volumetrics and Morphology by CMR

	**No aspirin**	**Aspirin**	***p* value**
LVEDV (ml), mean (SD)	148 (41)	146 (35)	0.84
LVESV (ml), mean (SD)	63 (22)	61 (21)	0.57
LVEF (%), mean (SD)	58 (8)	59 (7)	0.42
CI (l/min/m^2^), mean (SD)	2.6 (0.5)	2.8 (0.7)	0.39
Global native T1 (ms), mean (SD)	1,050 (78)	1,044 (56)	0.69
Global ECV (%), mean (SD)	29 (4)	27 (4)	**0.04**
Late gadolinium enhancement (*n*)	7 (24%)	31 (29%)	0.61

CI, cardiac index; ECV, extraceullular volume; LVEDV denotes left ventricular end-diastolic volume; LVEF, left ventricular ejection fraction; LVESV, left ventricular end-systolic volume; SD, standard deviation.

Statistically significant p-values are shown in bold.

### Myocardial perfusion

**Figure 1:**
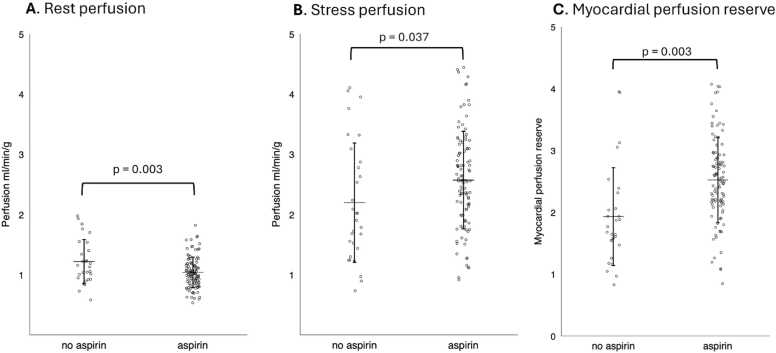
Box-and-whisker plots showing (A) global rest perfusion, (B) global stress perfusion, and (C) myocardial perfusion reserve (MPR) in patients receiving aspirin therapy compared with those not. The mean ± 1 SD is shown in each panel.

Resting perfusion was significantly lower in the aspirin group (1.0 ± 0.3 vs 1.2 ± 0.4 ml/min/g, *p* = 0.003). In contrast, stress perfusion was significantly higher in aspirin-treated patients (2.6 ± 0.8 vs 2.2 ± 1.0 ml/min/g, *p* = 0.037). Consequently, global MPR was significantly higher in patients receiving aspirin therapy (2.5 ± 0.7 vs 1.9 ± 0.8, *p* < 0.001), as illustrated in Figure 1.

Similar results were seen when rest RPP corrected values were used for rest perfusion (1.1 ± 0.3 vs 1.2 ± 0.4 ml/min/g, *p* = 0.038) or MPR (2.5 ± 0.8 vs 2.0 ± 0.9, *p* = 0.004).

MP for CAV 0 to 1 patients also demonstrated similar results: rest perfusion (1.0 ± 0.2 vs 1.2 ± 0.4 ml/min/g, *p* = 0.003), stress perfusion (2.6 ± 0.8 vs 2.3 ± 1.0 ml/min/g, *p* = 0.044) and MPR (2.6 ± 0.7 vs 2.0 ± 0.8, *p* < 0.001).

### Uni- and multivariable analysis

Univariate linear regression demonstrated a significant association between MPR and aspirin therapy (coefficient 0.59, *p* < 0.001), time from HTx to CMR (coefficient −0.036, *p* < 0.001) and CAV grade (coefficient −0.49, *p* < 0.001), see Table 3. In the multivariable model adjusting for sex, years from HTx to CMR, CAV grade, diabetes, statin therapy, and everolimus therapy, aspirin therapy remained significantly associated with higher MPR (coefficient 0.45, *p* < 0.01) and CAV grade was inversely associated with MPR (coefficient −0.40, *p* < 0.01).

**Table 3:** Uni- and Multivariable Linear Regression for Predictors of Myocardial Perfusion Reserve

	**Univariate**		**Multivariate**	
**Variable**	**Coefficient**	***p* value**	**Coefficient**	***p* value**
Aspirin	**0.59**	**<0.001**	**0.44**	**0.01**
Sex	0.08	0.61	**−**0.05	0.72
Time from HTx to CMR (years)	**−0.04**	**<0.001**	**−**0.01	0.19
CAV grade	**−0.49**	**<0.001**	**−0.40**	**<0.01**
Post transplantation diabetes	**−**0.29	0.06	**−**0.18	0.21
Statin treatment	0.26	0.33	0.07	0.77
Everolimus treatment	0.03	0.84	0.10	0.47

Statistically significant p-values are shown in bold.

## Discussion

In this retrospective cohort of heart transplant recipients undergoing routine surveillance CMR and coronary angiography, aspirin therapy was independently associated with higher MPR. Patients treated with aspirin had lower resting perfusion, higher stress perfusion, and consequently a higher global MPR compared with patients not receiving aspirin. The association between aspirin use and higher MPR remained significant after multivariable adjustment for factors known to influence CAV and microvascular function, including time from transplantation, diabetes, statin therapy, and everolimus use. NT-proBNP was higher in the non-aspirin group; however, no other differences were seen in laboratory tests. Together, these findings suggest that aspirin therapy may be linked to more preserved coronary vasodilatory capacity and potentially less advanced microvascular involvement of CAV.

### Aspirin use and CAV

Previous studies have primarily evaluated aspirin use after heart transplantation in relation to clinical outcomes and angiographically defined CAV.^7^, ^8^, ^9^ In contrast, the present study extends these observations by using fully quantitative CMR perfusion mapping as a functional marker of coronary microvascular and vasodilatory function. Rather than focusing solely on the presence or absence of angiographic stenoses, we assessed global and regional perfusion and MPR, which are sensitive markers of both epicardial and microvascular disease burden.^10^, ^11^, ^12^, ^13^, ^22^, ^23^, ^24^ The finding that aspirin therapy is associated with higher MPR, even after adjustment for key clinical and pharmacologic covariates, supports the hypothesis that aspirin may exert beneficial effects at the level of the coronary microcirculation in the transplanted heart.

### Myocardial perfusion and the assessment of CAV

Quantitative stress perfusion CMR has emerged as a tool for the non-invasive assessment of CAV, capable of detecting microvascular dysfunction and diffuse vasculopathy that may be missed by conventional angiography.^12^, ^13^, ^14^, ^19^, ^20^ Prior CMR studies in heart transplant recipients have demonstrated that reduced MPR is associated with the presence and severity of CAV and impaired graft function.^10^, ^11^, ^12^, ^13^, ^19^, ^20^

Our results demonstrate the use of quantitative perfusion mapping as a functional endpoint in pharmacologic and mechanistic studies in heart transplantation. By demonstrating an association between aspirin therapy and higher MPR, our study illustrates how CMR-based perfusion metrics can be used to interrogate the potential vascular effects of commonly used post-transplant therapies. Interestingly, ECV was significantly lower in patients with aspirin therapy and a possible link to higher MPR needs to be further examined. The observed differences in MPR occurred in the absence of significant differences in left ventricular volumetrics or presence of LGE, underscoring that perfusion mapping provides information that is not captured by standard functional and morphological parameters.

### Pathophysiological considerations

The mechanisms by which aspirin might influence MP and MPR in the transplanted heart are likely multifactorial. Aspirin’s primary antiplatelet effect—through irreversible inhibition of cyclo-oxygenase-1 and suppression of thromboxane A₂—reduces platelet aggregation and microthrombus formation, which may be particularly relevant in the setting of diffuse intimal hyperplasia and microvascular narrowing characteristic of CAV.^1^, ^7^ By limiting platelet activation, aspirin could mitigate microvascular obstruction and improve downstream perfusion during hyperemia.

Beyond platelet inhibition, aspirin has been reported to modulate endothelial function and exert anti-inflammatory effects, including decreased expression of adhesion molecules and reduced inflammatory cytokine signaling. In the allograft setting, where chronic immune-mediated endothelial injury and low-grade inflammation drive CAV progression, such effects may translate into better preserved microvascular reactivity and structural integrity.^5^, ^7^ The association of aspirin therapy with higher MPR, a marker that integrates both epicardial and microvascular vasodilatory capacity, is consistent with these proposed mechanisms.

Pharmacological therapies also play a critical role in modulating the development and progression of CAV. Statins are widely used and have demonstrated benefits through lipid-lowering, anti-inflammatory, and immunomodulatory effects.^1^, ^6^ Proliferation signal inhibitors, such as everolimus and sirolimus, reduce intimal thickening and may attenuate CAV progression^25^ but have not resulted in improved long-term clinical outcomes.

An interesting observation in our cohort was that resting perfusion was slightly lower, whereas stress perfusion and MPR were higher in patients receiving aspirin. We emphasize that lower resting perfusion alone should not be interpreted as a favorable marker of vascular health, as resting flow is influenced by myocardial oxygen demand, heart rate, blood pressure, loading conditions, and autoregulatory tone. Notably, prior work in transplanted hearts has demonstrated that reduced coronary flow reserve is often driven by elevated resting flow rather than impaired stress flow, suggesting that higher resting perfusion may reflect abnormal microvascular regulation rather than better vascular health.^26^ Accordingly, lower resting perfusion in the presence of higher stress perfusion and preserved vasodilatory reserve may be consistent with more intact autoregulatory function. The more relevant finding in our study is therefore the higher stress perfusion and preserved vasodilatory reserve reflected by the higher MPR, which suggests better preserved coronary microvascular function in the aspirin-treated group. The persistence of this pattern after rate-pressure-product correction supports that the findings are not solely attributable to baseline hemodynamic differences, although residual confounding cannot be excluded.

### Clinical implications

Our findings have several potential clinical implications. It has previously been suggested that aspirin therapy may be linked to macrovascular outcomes, such as angiographic CAV or clinical events.^7^, ^8^, ^9^ Our findings further suggest a possible association with better microvascular function, as reflected by higher MPR, a measure linked to coronary microvascular function.^10^, ^27^ This supports the continued use of aspirin in post-transplant management, particularly considering current guideline re--ations that assign only a Class IIb indication for its use in CAV prevention.^4^ While our observational data cannot establish causality, the association between aspirin and higher MPR provides imaging-based support for a potential protective vascular effect in the transplanted heart. The ongoing Antiplatelet Therapy in Heart Transplantation (AERIAL) Trial (NCT04770012) may provide a more definitive answer to the question if aspirin can prevent the development of CAV. Interestingly, this study is a 3-arm randomized comparison of aspirin, clopidogrel, and placebo and will thus also investigate if the anti-inflammatory properties of aspirin are important in the pathogenesis of CAV.

Second, the study highlights the value of quantitative stress perfusion CMR as a tool to investigate the functional impact of common post-transplant medications. CMR-derived MPR may serve as a sensitive intermediate endpoint in future trials evaluating novel antiplatelet, anti-inflammatory, or immunomodulatory strategies aiming to attenuate CAV progression.

### Limitations

Several limitations of this study should be acknowledged. First, the retrospective observational design precludes any causal inference regarding the effect of aspirin on MP or CAV; unmeasured or residual confounding may partly account for the observed associations. Aspirin use was not randomized, and treatment decisions may have been influenced by factors such as perceived cardiovascular risk, comorbidities, or center-specific practice patterns.

We acknowledge that discontinuation of aspirin is not random and may reflect clinical factors such as bleeding risk or comorbidity, which could also contribute to residual confounding.

Second, baseline differences between the aspirin and non-aspirin groups may reflect underlying clinical heterogeneity, including differences in time from transplantation, age, NT-proBNP levels, and concomitant therapies. Although we adjusted for key clinical variables and concomitant therapies known to influence CAV (including time from transplantation, diabetes, statin therapy, and everolimus use), other potential confounders—such as donor characteristics, detailed rejection history, or cumulative immunosuppressive exposure—were not fully captured. The aspirin group also had more frequent use of certain immunosuppressive agents and statins, which could contribute to the observed perfusion differences despite statistical adjustment.

Third, CAV grading was based on invasive coronary angiography without systematic intravascular imaging or physiological assessment, which may underestimate early or predominantly microvascular disease. Nonetheless, this reflects current routine clinical practice at many centers and is consistent with ISHLT grading re--ations.^4^

Fourth, the study does not investigate the clinical implication of reduced MPR, which is ultimately the question to be addressed.

Finally, the study cohort, while relatively large for a CMR-based transplant series, remains modest in size and imbalanced between aspirin and non-aspirin groups, potentially limiting statistical power for subgroup analyses. To this point, we did not use propensity score matching that would substantially reduce the effective sample size and statistical power, potentially introducing additional bias. The findings should therefore be confirmed in larger, ideally multicenter, cohorts and in prospective studies with standardized aspirin protocols.

## Conclusion

In this retrospective study, aspirin therapy in heart transplant recipients was independently associated with higher MPR, suggesting an association with better preserved coronary vasodilatory capacity. These findings support the utility of quantitative CMR perfusion mapping as a sensitive marker of microvascular dysfunction and therapeutic response in the post-transplant population.

## Declaration of generative AI and AI-assisted technologies in the manuscript preparation process

During the preparation of this work the authors used ChatGPT v5.2 (OpenAI) to assist with language editing and improvement of grammar. After using this tool, the authors reviewed and edited the content as needed and take full responsibility for the content of the published article.

## Disclosure statement

No disclosures.

## Data availability statement

The data underlying this article will be shared on reasonable request to the corresponding author.

## Financial support

O.Ö.B. was supported by grants from the Swedish Heart and Lung Foundation, USVE-grant, Ulla Ekdahls stiftelse, Hjelms stiftelse, and Hains stiftelse.

## Declaration of Competing Interest

The authors declare the following financial interests/personal relationships, which may be considered as potential competing interests: Oscar Braun reports financial support was provided by the Heart and Lung Foundation. Oscar Braun reports financial support was provided by USVE grant. Oscar Braun reports financial support was provided by Ulla Ekdahls stiftelse. Oscar Braun reports financial support was provided by Hjelms Stiftelse. Oscar Braun reports financial support was provided by Hains Stiftelse. If there are other authors, they declare that they have no known competing financial interests or personal relationships that could have appeared to influence the work reported in this paper.
